# The Effect of Puberty on Interaction between Vitamin D Status and Insulin Resistance in Obese Asian-Indian Children

**DOI:** 10.1155/2012/173581

**Published:** 2012-08-16

**Authors:** Rajesh Khadgawat, Tushanth Thomas, Monita Gahlot, Nikhil Tandon, Vin Tangpricha, Deepak Khandelwal, Nandita Gupta

**Affiliations:** ^1^Department of Endocrinology and Metabolism, All India Institute of Medical Sciences, New Delhi 110029, India; ^2^Department of Dietetics and Nutrition, All India Institute of Medical Sciences, New Delhi 110029, India; ^3^Division of Endocrinology, Metabolism and Lipids, Department of Medicine, Emory University School of Medicine, Atlanta, GA 30322, USA

## Abstract

To study the effect of puberty on the relationship between serum 25-hydroxyvitamin D (25(OH)D) and parameters of insulin kinetics in obese Asian-Indian children. *Material and Methods*. The study population included 62 obese Asian-Indian children and adolescents in the age group of 6–17 years. Blood glucose, serum insulin, and serum 25(OH)D were measured. Total body fat was measured by dual energy X-ray absorptiometry. Indices of insulin resistance (HOMA-IR, AUC for insulin) and sensitivity (WBISI) were calculated after oral glucose tolerance test. *Result*. A total of 62 subjects (35 boys; mean age = 13.0 ± 3 years; BMI = 29.3 ± 4.8 kg/sq M; 19 subjects in Tanner stage 1, 11 in stage 2, 6 in stage 3, 3 in stage 4, and 23 subjects in Tanner stage 5) were studied. All study subjects were vitamin D deficient with a mean serum 25(OH)D of 8.5 ± 4.2 ng/mL. No significant relationship was observed between serum 25(OH)D and parameters of insulin kinetics in prepubertal children. However, a significant inverse correlation was seen between serum 25(OH)D and HOMAIR (*r* = −0.41, *P* = 0.03) in postpubertal subjects. *Conclusion*. The relationship between vitamin D status and parameters of insulin kinetics are affected by puberty.

## 1. Introduction

Vitamin D deficiency was once thought to exclusively affect bone metabolism, but now there is ample evidence of its role in many other conditions including metabolic syndrome, autoimmune diseases, and cancer [1]. Vitamin D receptors are recognized to be in numerous extraskeletal tissues, such as pancreas and muscle [2]. A systematic review by Pittas and colleagues [3] reported that vitamin D may have a beneficial effect on the action of insulin, either directly or indirectly. Several observational studies in adults, including the Framingham Heart Study [4], have reported an inverse association between vitamin D status and insulin resistance. However, data from children and adolescents do not consistently report this inverse relationship [5–10]. Even in studies reporting this association in adolescents and children, the strength of association has been found to be very modest [6]. Further, none of the studies in adolescents have studied the effect of puberty on this association. A fall in insulin sensitivity with compensatory increase in insulin secretion has been reported in puberty [11, 12].

High prevalence of cardiometabolic risk factors, including insulin resistance (64.8% of normal weight children had at least one cardiometabolic abnormality), even in healthy young children of normal weight, has been reported in Indian children and adolescents [13]. South-Asian adolescents, including those with normal BMI, have a higher prevalence of insulin resistance when compared with age and BMI matched European adolescents [14, 15]. This ethnic difference in insulin sensitivity was attributed to the higher body fat percentage in South-Asian children [15]. An alternative hypothesis to explain the ethnic differences in insulin sensitivity is vitamin D deficiency. Despite plenty of sunlight available throughout the year, a high prevalence of vitamin D deficiency has been reported in all age groups including toddlers, school children, pregnant women and their neonates, and adult males and females residing in rural and urban India [16]. Vitamin D deficiency has been reported in 84–92% children in the age group of 10–18 [17].

In view of high prevalence of both, insulin resistance and vitamin D deficiency in Asian-Indian children and adolescents, we sought to examine the relationship between vitamin D status and insulin resistance. We also sought to determine if puberty affected the relationship of vitamin D status and parameters of insulin resistance and sensitivity in Asian-Indian children and adolescents.

## 2. Material and Methods

### 2.1. Study Design

The study was conducted as a prospective observational study after approval from the Ethics Committee of the All India Institute of Medical Sciences (AIIMS), New Delhi. Subjects were selected from the children and adolescents attending the endocrinology outdoor services at AIIMS for evaluation of obesity. Written informed consent was obtained from parents of all subjects. Additionally, assent was also obtained from subjects more than seven years old. The inclusion criteria included children between the ages of 6–17 years with obesity as per International Obesity Taskforce (IOTF) criteria [18]. The exclusion criteria included subjects with diagnosed diabetes mellitus or taking metformin or any weight reducing drugs, subjects with any known systemic illness or endocrine or metabolic disorders, known to be associated with obesity, or subjects with symptoms to suggest hypothalamic obesity were excluded from the study.

### 2.2. Methods

Detailed history, clinical and anthropometric examination was carried out. Blood samples were collected in fasting state (minimum 9 hours fasting), followed by oral glucose tolerance test (anhydrous glucose = 1.75 gm/kg bodyweight, maximum of 75 gram, dissolved in 250 mL of water, ingested over 5 minutes) with sample collection at 60 and 120 minutes after glucose ingestion. 

### 2.3. Anthropometric Measurements

All measurements were made with subjects dressed in minimal light clothing but without footwear. Height was measured with Holtain's stadiometer (Holtain Inc., Crymych, Pembs. UK). Weight was measured with the same digital weighing machine. Waist circumference was measured (nonelastic measuring tape) at the end of normal expiration at the midpoint between the iliac crest and the lower edge of ribs in the midaxillary line. Hip circumference was measured at the point of maximum circumference over the buttocks. Blood pressure (BP) was measured in the right upper limb in sitting position with a mercury sphygmomanometer after 5-minute rest with an appropriate size cuff. BP was measured three times and mean value was taken. Total body fat was measured with dual energy X-ray absorptiometry (DXA, Hologic QDR 4500 with pediatric software, Hologic Inc., Waltham, MA, USA). Fat mass index (FMI, fat mass in kg/height in meter^2^) and fat-free mass index (FFMI, lean body mass+ BMC)/(height in meter)^2^ were calculated. Bone mineral area, content (BMC) and areal density (aBMD) at the femoral neck, lumbar spine (L1-5), and forearm were measured, using a Hologic QDR 4500A fan beam DXA machine. Hologic spine phantom (Hologic spine phantom number 21373) was scanned daily before subject evaluation. The measured phantom bone mineral density was stable throughout the study period at 0.915–0.945 gm/cm^2^. Similarly, whole body phantom (Hologic WB number 1252) was also scanned before subject evaluation and remained stable during study period. The *in vivo* precision error for adults was 0.62% for femoral neck aBMD, 0.65% for lumbar spine aBMD, and 0.77% for forearm aBMD. However, in view of additional radiation exposure, the reproducibility of these scans was not assessed among children.

### 2.4. Pubertal Staging

Pubertal stage was assessed by a single endocrinologist, based on breast stage and pubic hair development in girls [19] and genitalia development in boys [20].

### 2.5. Measurement of Carotid Intima Media Thickness (CIMT)

B-mode ultrasonography was used to measure CIMT (7.5–10 MHz probe, Philips Envisor ultrasound machine) using standard protocol [21]. 

### 2.6. Indices of Glucose and Insulin Metabolism

- Insulin Resistance (IR) was calculated by computer-based model called homeostasis assessment model (HOMA-IR) utilizing fasting blood glucose and fasting serum insulin levels [22].
- The Whole Body Insulin Sensitivity Index (WBISI) or Matsuda Index was calculated by the formula suggested by Matsuda and DeFronzo [23].
- Area under Curve (AUC) was calculated for blood glucose (0, 60, and 120 minutes) and serum insulin (0, 60, and 120 minutes) by using trapezoidal rule.

### 2.7. Biochemical Analysis

Complete blood counts, liver function tests, renal function test, serum calcium (corrected calcium with serum albumin), phosphate, alkaline phosphatase, uric acid, and blood glucose were measured in all subjects with an automated chemistry analyzer (Roche Hitachi 912 Chemistry Analyzer, GMI Inc., USA). Glycosylated hemoglobin (HbA1c) was measured in whole blood using ion-exchange high performance liquid chromatography (Bio-Rad Laboratories Inc., CA, US). Serum insulin was measured on an autoanalyzer (Roche Elecsys 2010) using electrochemiluminometric assay. Serum total cholesterol (TC), triglyceride (TG), and HDL cholesterol (HDL-C) levels were estimated directly with automated analyzer, while LDL cholesterol (LDL-C) was estimated by using the Friedewald equation [24]. Serum 25(OH)D was measured with radioimmunoassay (RIA, DiaSorin Inc., Stillwater, USA). Our laboratory is registered with vitaminD externalquality assessment scheme (http://www.deqas.org/). Vitamin D deficiency was defined as a serum 25(OH)D levels less than 20 ng/mL. This was further subdivided to severe, moderate, and mild vitamin D deficiency if serum 25(OH)D levels were <5 ng/mL, 5–<10 ng/mL, and 10–<20 ng/mL respectively [25].

## 3. Statistical Analysis

Statistical analysis was carried out using SPSS (SPSS version 11.5; SPSS Inc., Chicago, IL). Descriptive statistical analyses were used for demographic information. Results were expressed as mean ± standard deviation (SD) unless otherwise specified. Continuous variables not showing normal distribution were treated with appropriate log transformations before any parametric analysis. Student's *t*-test was used to determine statistical difference between subjects with serum 25(OH)D <10 ng/mL or more. Pearson's correlation was used to assess association between continuous variables and partial correlations were used to determine relationship after adjusting for relevant covariates. Bonferroni's corrections were used for multiple comparisons. Stepwise linear regression analysis was used with serum 25(OH)D as dependent variable in the model. Values for serum 25(OH)D, serum insulin, HOMA-IR, and Matsuda ISI were not normally distributed; therefore, they were logarithmically transformed which yielded normal distribution.

## 4. Results

A total of 62 subjects participated in this cross-sectional study from June 2008 to May 2009. The mean age of subjects was 13.0 ± 3 years (35 boys; 27 girls). Assessment of pubertal status by Tanner's method showed 19 subjects in stage 1, 11 in stage 2, 6 in stage 3, 3 in stage 4, and 23 subjects in puberty stage 5. Descriptive details of study subjects are given in Table 1. No significant difference was observed between boys and girls except higher WHR in boys than girls (0.93 ± 0.05 versus 0.89 ± 0.05; *P* = 0.013).

### 4.1. Serum 25-Hydroxyvitamin D (25(OH)D) Concentrations

All study subjects were classified as vitamin D deficient (mean ± SD 8.5 ± 4.2 ng/mL; maximum–minimum: 3.9–19.2 ng/mL, median = 6.9 ng/mL). Severe vitamin D deficiency (<5 ng/mL) was seen in 11 subjects (17.7%) while 30 subjects had serum 25(OH)D levels between 5–<10 ng/mL (48.3%). Serum 25(OH)D equal to or more than 10 but less than 20 ng/mL was present in 21 subjects (33.8%). No significant difference was seen in serum 25(OH)D concentrations between boys and girls.

**Figure 1:**
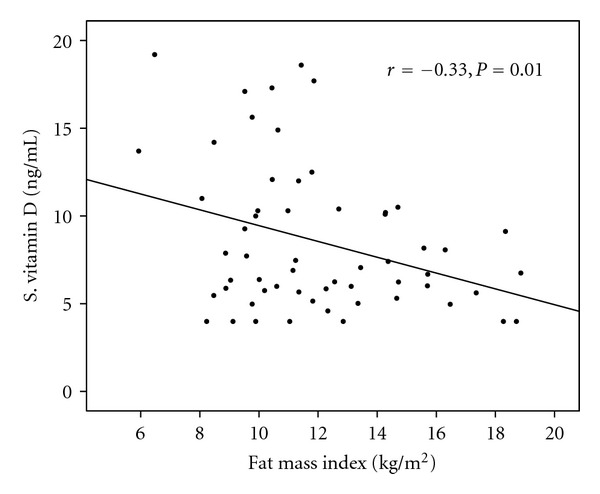
Correlation between Serum 25-hydroxyvitamin D concentration and fat mass index (FMI) in all subjects.

Pearson correlation coefficient analyses showed significant inverse relationship between serum 25(OH)D and total body fat percentage (*r* = −0.31, *P* = 0.01) and serum 25(OH)D and FMI (*r* = −0.33, *P* = 0.01, Figure 1). Partial correlation analysis showed persistence of significant correlation between serum 25(OH)D and total body fat percentage (*r* = −0.37, *P* = 0.005) and between serum 25(OH)D and FMI (*r* = −0.36, *P* = 0.006) even after adjustment for age, sex, pubertal stage, and BMI. Although blood glucose and serum insulin levels showed inverse relationship trends with serum 25(OH)D, but these were not statistically significant (data not shown). Similarly, no statistically significant correlation was seen between serum 25(OH)D and parameters of insulin sensitivity or resistance.

### 4.2. Body Fat and Insulin Resistance

FMI showed positive correlation with HOMA-IR (*r* = 0.26; *P* = 0.04), AUC insulin (*r* = 0.41; *P* = 0.001), AUC glucose (*r* = 0.31; *P* = 0.018), and negative correlation with WBISI (*r* = −0.42; *r* = 0.001). However, total body fat did not show any correlation with parameters of glucose and insulin parameters. 

On the basis of serum 25(OH)D, subjects were divided into two groups, group A with serum 25(OH)D level <10 ng/mL (*n* = 41) and group B with serum 25(OH)D level ≥10 ng/mL (*n* = 21) (an arbitrary cutoff to compare subjects with mild vitamin D deficiency with moderate and severe vitamin D deficiency, 25). Descriptive details of these two groups are provided in Table 1. No statistical significant difference was seen in blood glucose, serum insulin, and AUC for both, glucose and insulin between group 1 and group 2. Trends for higher insulin resistance (HOMA-IR) and lower insulin sensitivity (WBISI) were seen in subjects with lower serum 25(OH)D concentrations but not statistically significant.

Total body fat percentage and FMI were significantly higher in group 1 in comparison to group 2 (*P* = 0.015 and 0.007, resp.). However, no significant difference was seen in waist and hip circumference and waist hip ratio. Similarly, FFMI, lipid parameters, or thyroid profile were also not different between the two groups.

### 4.3. Effects of Pubertal Stage

Subjects were divided into three groups according to their pubertal status. Group 1 (prepubertal group; Tanner stage 1) had 19 subjects, group 2 (peripubertal group; Tanner stage 2, 3, and 4) had 20, while group 3 (postpubertal group; Tanner stage 5) had 23 subjects. No significant correlation was seen between vitamin D status and any other parameters including total body fat percentage, FMI and parameters of insulin resistance and sensitivity in the prepubertal (group 1) or peripubertal groups (group 2). Partial correlations after adjusting for age, sex, and BMI were also nonsignificant for both groups (data not shown).

**Figure 2:**
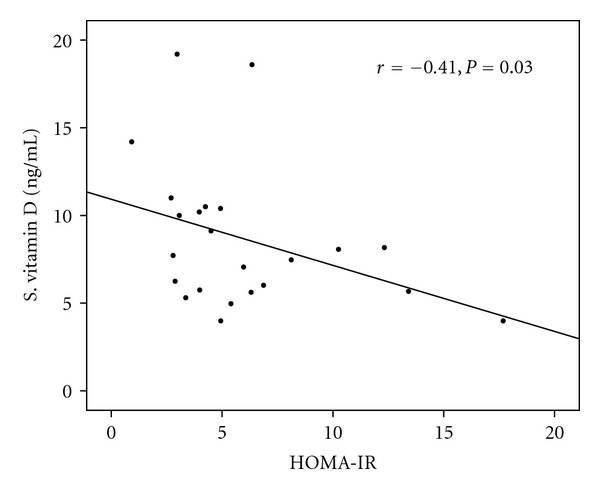
Correlation between serum 25(OH)D concentrations and HOMA-IR in post-pubertal subjects (group 3).

In postpubertal subjects (group 3, *n* = 23), a significant inverse correlation was seen between serum 25(OH)D and HOMA-IR (*r* = −0.41, *P* = 0.03, Figure 2). This association between serum 25(OH)D and HOMA-IR persisted even after control for age, sex, and BMI (*r* = −0.505, *P* = 0.023). Positive trends of association between serum 25(OH)D and WBISI were also seen, but this was not significant (*r* = 0.29, *P* = 0.17), even after adjusting for age, sex, and BMI (*r* = 0.35, *P* = 0.13). Serum 25(OH)D showed significant inverse correlation with total body fat percentage (*r* = −0.58, *P* = 0.003) and FMI (*r* = −0.49, *P* = 0.016) which improved after adjusting for age sex and BMI (*r* = −0.63, *P* = 0.002 for total body fat percentage; *r* = −0.582,  *P* = 0.007 for FMI). Serum 25(OH)D also showed significant inverse relationship with fasting serum insulin levels (*r* = −0.44, *P* = 0.034) which improved after adjusting for age, sex, and BMI (*r* = −0.53, *P* = 0.016).

When prepubertal subjects (group 1) were compared with the prepubescent subjects (group 2), no significant difference was seen in any of the parameters including blood glucose, serum insulin, HOMA-IR, and WBISI. Similarly, when prepubertal subjects were compared with all other subjects (subjects in group 2 and 3 combined), significantly higher insulin sensitivity (WBISI, mean ± SD 6.07 ± 5.7 versus 3.42 ± 2.7, *P* < 0.01) and lower insulin resistance (HOMA-IR, mean ± SD 3.68 ± 2.7 versus 5.22 ± 4.0, *P* = 0.04) were seen in prepubertal subjects.

## 5. Discussion

This is the first study examining the relationship between serum 25(OH)D levels and parameters of insulin resistance in obese Asian-Indian children. Our study confirms a significant inverse relationship between serum 25(OH)D levels and body fat indices as reported in other studies in children [6, 10]. We demonstrated trends for that higher serum 25(OH)D levels are associated with better insulin sensitivity and less insulin resistance though this correlation did not reach statistical significance. One of the main reasons for the absence of significance could be the universal presence of vitamin D deficiency (<20 ng/mL) resulting in very narrow range of serum 25(OH)D. We also show, for the first time, that the association between serum 25(OH)D and insulin resistance and sensitivity is influenced by pubertal staging. When subjects were stratified by pubertal stage, only subjects in Tanner stage 5 exhibited a significant correlation between serum 25(OH)D and HOMA-IR, while prepubertal subjects did not show statistically significant correlation.

High prevalence of vitamin D deficiency has been reported across the age range in pediatric, adolescent, and adult in obese individuals [5, 6, 26–31]. All our study subjects had vitamin D deficiency. This may be just reflection of very high prevalence of vitamin D deficiency in Asian-Indians [16, 17]. However, high prevalence of vitamin D deficiency has been reported in obese children and adolescents from populations where vitamin D deficiency is not that prevalent [5, 6, 10]. Reis et al., in a study of 3577 adolescents in the age group of 12–19 years, found significantly lower serum 25(OH)D levels in adolescents with BMI > 95th centile in comparison to subjects with BMI < 95th centile (*P* < 0.001) [7]. It has been postulated that increased sequestration of vitamin D in fat tissues, leading to decreased vitamin D bioavailability, low dietary vitamin D intake due to poor nutritional habits, and minimal sun exposure due to sedentary indoor lifestyle are important factors associated with high prevalence in the obese population [29, 32]. It has also been suggested that vitamin D deficiency is a cause of common obesity and can account for the secular trends in the prevalence of obesity and for individual differences in its onset and severity [33].

The inverse relationship between serum 25(OH)D and insulin resistance in adults have been reported in most studies, though some have failed to find this association [3]. In comparison, reports in children and adolescents have shown conflicting results. Delvin et al. [6], in a study of 1745 French-Canadian children, reported modest but significant negative associations between serum 25(OH)D and HOMA-IR. Each 10-nmol/L (4 ng/mL) increase in serum 25(OH)D was associated with lower glycemia and HOMA-IR [6]. Reinehr et al. [8] in a study of 133 obese Caucasian children did not find significant correlation between serum 25(OH)D levels and insulin, HOMA-IR and HOMA-B %. In another study based on NHANES data of 3577 adolescents from 2001–2004 showed that serum 25(OH)D levels were inversely associated with plasma glucose concentration (*P* = 0.01). Lenders et al., in a study of 58 obese adolescents, did not found any correlation between insulin indices and serum 25(OH)D in both, adjusted and unadjusted models. In our study, we did not find a statistically significant association between serum 25(OH)D and parameters of insulin sensitivity and resistance. However, trends for higher blood glucose and serum insulin levels were seen in subjects with low serum 25(OH)D level. Similar observation was also seen with AUC glucose and AUC insulin. No significant difference was seen in insulin resistance and sensitivity between group 1 and group 2. This is in contrast to the observations made by Ashraf et al. who reported in 51 African-American obese female adolescents [10] that higher serum 25(OH)D concentrations were associated with significantly higher WBISI (*P* = 0.018) but no difference in HOMA-IR. The AUC for insulin was also higher in subjects with lower serum 25(OH)D. These differences may be due to marked difference in severity of obesity as mean BMI of study subjects was 43.3 ± 9.9 in study by Ashraf et al., while it was 29.3 ± 9.8 kg/sqM in our study subjects. Studies in adult population have shown that parameters of insulin sensitivity and insulin resistance as calculated by hyperinsulinemic euglycemic clamp study (*M* value) correlated better with serum 25(OH)D levels than indirect parameters like HOMA-IR [34]. Studies with direct measures of insulin sensitivity like clamp studies are required to further investigate these issues in pediatric and adolescent population.

Insulin resistance is a feature of obesity and hallmarks normal pubertal development. The phenomenon of pubertal insulin resistance has been well described in cross-sectional as well as longitudinal studies [35]. A longitudinal study of insulin resistance in American children showed that during puberty, insulin sensitivity decreased by *∼*50%, with a compensatory increase in plasma insulin which is independent of the changes in body fat [36]. These changes are thought to be mediated by interaction of various hormones during puberty including increased growth hormone secretion [36]. No studies have yet reported the relationship between serum 25(OH)D concentrations and insulin sensitivity and resistance in pediatric and adolescent population and the potential influence of puberty except one [37]. Lenders et al. reported no significant association between serum 25(OH)D and insulin indices, even after adjusting for Tanner stage [37]. However, our study, for the first time, shows that the relationship between serum 25(OH)D concentrations and insulin resistance is influenced by puberty. When subjects with all stages of puberty were analyzed, no significant correlation was found, but when subjects in Tanner stage 5 were analyzed alone, significant inverse correlation was seen between serum 25(OH)D concentrations and HOMA-IR (*r* = −0.41, *P* = 0.03). This association persisted/improved after controlling for age, sex, and BMI (*r* = −0.505, *P* = 0.023) but disappeared when subjects were controlled for FMI (*r* = − 0.27, *P* = 0.24). These differences in results may be due to differences in study population characteristics. 

Most of the evidence of an association between adiposity and vitamin D status comes from adult studies [4, 33]. A recently published study [38] of 382 healthy subjects aged 6–21 years with DXA scans showed that serum 25(OH)D concentrations were more likely to be lower in those with greater BMI *z*-scores and FM, but not FFM. When this association was adjusted for possible covariates in the model, it became nonsignificant. Using DXA to measure FM, Lenders et al. [37] showed that obese adolescents with and without vitamin D deficiency differed according to FM and FM percentage, but not lean mass and this relation of FM percentage to serum 25(OH)D remained significant after potential confounding variables were adjusted for. Rajakumar et al. showed that vitamin D deficiency was associated with higher visceral adipose tissue in white and greater subcutaneous adipose tissue in black American children and adolescents [39]. In agreement with these studies, our findings indicate that body fat indices (total body fat percentage and FMI) are inversely correlated with serum 25(OH)D level. This correlation persisted even after adjustment for possible covariates like age, sex, and BMI. The differences in study findings may be explained in part by differences in study design and underlying sample characteristics like BMI and variation in serum 25(OH)D concentrations.

The major strengths of our study are the use of robust measures of insulin sensitivity and resistance based on OGTT rather than fasting sample and all our study subjects belonged to one ethnic group. We have used DXA for assessment of body fat as opposed to several studies which have used BMI as a surrogate for body fat. The limitations of our study included, small sample size, presence of vitamin D deficiency in all study subjects which gave us very narrow range of serum 25(OH)D concentrations thereby limiting comparisons. Absence of PTH measurement was another important limitation, since it has been implicated in pathogenesis of insulin resistance and metabolic syndrome. Another important limitation was inability of DXA technique to differentiate between subcutaneous and visceral adipose tissue.

In conclusion, our study demonstrates a significant inverse relationship in obese Asian-Indian children between body fat indices and serum 25(OH)D concentrations which persists even after adjustment of covariates. The association between serum 25(OH)D and parameters of insulin sensitivity and resistance is influenced by puberty as a significant association was found between these two variables in postpubertal subjects (Tanner stage 5) only which remained significant even after adjustment for covariates. As association is not equal to causation, future longitudinal research studies are required in determining role of puberty on association between serum 25(OH)D and insulin resistance in both, obese as well as subjects with normal BMI.

## Figures and Tables

**Table 1:** Descriptive characteristics of study population.

Parameter	Mean ± SD	Group A: serum 25(OH)D < 10 ng/mL	Group B: serum 25(OH)D ≥ 10 ng/mL	*P* value
Number of subjects	62	41	21	
Age (in yrs)	13.0 ± 3.1	12.6 ± 3.1	13.7 ± 3.1	0.2
BMI (Kg/M^2^)	29.3 ± 4.8	29.3 ± 5.2	29.3 ± 3.8	0.97
Waist circumference (cms)	89.6 ± 13.3	89.4 ± 15	90 ± 11.0	0.87
Hip circumference (cms)	97.8 ± 13.03	97.4 ± 13	99 ± 14	0.71
Waist hip ratio	0.92 ± 0.06	0.91 ± 0.06	0.91 ± 0.07	0.86
Systolic BP (mm of Hg)	120 ± 12	118 ± 12	124 ± 12	0.06
Diastolic BP (mm of Hg)	79 ± 8	78 ± 7	80 ± 9	0.55
Total cholesterol (mg/dL)	154 ± 30	155 ± 29	154 ± 34	0.94
Triglyceride (mg/dL)	116 ± 47	113 ± 49	122 ± 42	0.50
HDL cholesterol (mg/dL)	40 ± 6	40 ± 6	39 ± 4	0.78
LDL cholesterol (mg/dL)	92 ± 25	92 ± 24	91 ± 28	0.86
S. T4 (*μ*g/dL)	9.44 ± 2.1	9.50 ± 2.2	9.31 ± 2.0	0.74
S. TSH (mIU/L)	3.79 ± 2.2	3.86 ± 2.4	3.65 ± 1.9	0.74
AST (IU/L)	34 ± 20	36 ± 24	30 ± 7	0.29
ALT (IU/L)	42 ± 40	46 ± 48	35 ± 14	0.32
Bl. glucose at 0 min (mg/dL)	95 ± 13	99 ± 17	93 ± 9	0.098
Bl. glucose at 60 min (mg/dL)	135 ± 33	141 ± 37	124 ± 22	0.06
Bl. glucose at 120 min (mg/dL)	120 ± 24	123 ± 26	114 ± 17	0.16
AUC glucose	241.7 ± 41.1	248.6 ± 45.7	231.3 ± 27.6	0.07
S. insulin at 0 min (mU/L)	20 ± 16	22 ± 18	15 ± 8	0.2
S. insulin at 60 min (mU/L)	120 ± 153	123 ± 118	118 ± 210	0.9
S. insulin at 120 min (mU/L)	120 ± 175	128 ± 162	105 ± 200	0.6
AUC insulin	185.8 ± 230.6	190.2 ± 185.1	177.1 ± 305.8	0.8
HOMA-IR	4.75 ± 3.7	5.17 ± 4.1	3.91 ± 2.5	0.2
WBISI	4.23 ± 4.0	4.05 ± 4.1	4.59 ± 3.7	0.39
HbA1c (%)	5.4 ± 0.4	5.4 ± 0.3	5.4 ± 0.5	0.93
Total body fat (%)	40.8 ± 6.6	42 ± 6.6	38.0 ± 5.7	0.015
Fat mass index (Kg/M^2^)	12.0 ± 3.1	12.7 ± 3.2	10.6 ± 2.3	0.007
Fat-free mass index (Kg/M^2^)	17.2 ± 2.9	17.0 ± 3.1	17.5 ± 2.4	0.53
Serum 25(OH)D (ng/mL)	8.5 ± 4.2	5.9 ± 1.4	13.6 ± 3.2	—
CIMT (mm)	0.06 ± 0.01	0.06 ± 0.01	0.05 ± 0.01	0.52

BMI: Body mass index, BP: blood pressure, AST: aspartate aminotransferase, ALT: alanine aminotreasferase, AUC: area under curve, WBISI: whole body insulin sensitivity index, CIMT: carotid intima media thickness.

## Abbreviations

BMI: Body mass in index
DXA: Dual energy X-ray absorptiometry
FMI: Fat mass index
FFMI: Fat-free mass index
HOMA-IR: Homeostasis assessment model-insulin resistance
WBISI: Whole body insulin sensitivity index
AUC: Area under curve
Serum 25(OH)D: Serum 25-hydroxyvitamin D.
